# Synthesis, Characterization, and Visible Light Curing Capacity of Polycaprolactone Acrylate

**DOI:** 10.1155/2018/8719624

**Published:** 2018-05-08

**Authors:** Jy-Jiunn Tzeng, Yi-Ting Hsiao, Yun-Ching Wu, Hsuan Chen, Shyh-Yuan Lee, Yuan-Min Lin

**Affiliations:** ^1^Department of Dentistry, National Yang-Ming University, No. 155, Sec. 2, Linong St., Beitou District, Taipei 112, Taiwan; ^2^Department of Stomatology, Taipei Veterans General Hospital, No. 201, Section 2, Shipai Road, Beitou District, Taipei City 112, Taiwan; ^3^Department of Dentistry, Taipei City Hospital, No. 145, Zhengzhou Rd., Datong District, Taipei 103, Taiwan

## Abstract

Polycaprolactone (PCL) is drawing increasing attention in the field of medical 3D printing and tissue engineering because of its biodegradability. This study developed polycaprolactone prepolymers that can be cured using visible light. Three PCL acrylates were synthesized: polycaprolactone-530 diacrylate (PCL530DA), glycerol-3 caprolactone triacrylate (Glycerol-3CL-TA), and glycerol-6 caprolactone triacrylate (Glycerol-6CL-TA). PCL530DA has two acrylates, whereas Glycerol-3CL-TA and Glycerol-6CL-TA have three acrylates. The Fourier transform infrared and nuclear magnetic resonance spectra suggested successful synthesis of all PCL acrylates. All are liquid at room temperature and can be photopolymerized into a transparent solid after exposure to 470 nm blue LED light using 1% camphorquinone as photoinitiator and 2% dimethylaminoethyl methacrylate as coinitiator. The degree of conversion for all PCL acrylates can reach more than 80% after 1 min of curing. The compressive modulus of PCL530DA, Glycerol-3CL-TA, and Glycerol-6CL-TA is 65.7 ± 12.7, 80.9 ± 6.1, and 32.1 ± 4.1 MPa, respectively, and their compressive strength is 5.3 ± 0.29, 8.3 ± 0.18, and 3.0 ± 0.53 MPa, respectively. Thus, all PCL acrylates synthesized in this study can be photopolymerized and because of their solid structure and low viscosity, they are applicable to soft tissue engineering and medical 3D printing.

## 1. Introduction

Biodegradable materials are drawing increasing attention in the field of 3D printing because they can be used to print body implants, tissue engineering scaffolds, and even drug-releasing capsule [1–3]. So far, polylactic acid (PLA) is one of the most common biodegradable materials used in 3D printing [4–6]. PLA can be easily heated above its melting temperature of 150°C–160°C and squeezed out from a nozzle in a 3D printer based on fused deposition modeling (FDM) [7, 8]. The major problem of FDM 3D printers is that they have low resolution compared with using stereolithography (SLA) or digital light processing (DLP) 3D printers [9, 10]. Another problem of FDM 3D printers is the slow printing speed because it forms a 3D object by stacking many thin material lines, consequently taking several hours to print a small object [11, 12]. This study developed biodegradable materials for use in DLP 3D printers in the near future.

The biodegradability of common polymers, such as polyglycolide (PGA), PLA, and polycaprolactone (PCL) polyesters, depends on their structures [13–15]. Their ester bonds undergo hydrolysis on reaction with water. Polyesters with longer alkyl backbone and more alkyl side chains are less hydrophilic and therefore have a longer degradation rate [16]. In general, PCL has a longer degradation rate than does PLA, and PLA shows a longer degradation rate than does PGA. Because of its longer degradation time, PCL has several potential tissue engineering and medical 3D printing applications.

PCL is a popular biodegradable material for resin additives, small scale modeling, and bone tissue engineering [17–22]. It can be synthesized by the ring-opening polymerization of *ε*-caprolactone [23, 24]. It has been prepared as scaffolds by using salt-leaching and thermally induced phase separation techniques. However, these two methods are inconsistent and therefore are unsuitable for largescale production.

The properties of PCL depend on its molecular weight [25, 26]. High-molecular-weight PCL (80 kDa) is a white solid with a *T*_g_ of 60°C. In contrast, low-molecular-weight PCL (0.53 kDa) is a transparent liquid at room temperature. For 3D printing applications, PCL must be kept in a liquid state; in other words, its molecular weight must be precisely controlled. In addition, low-molecular-weight PCL must contain functional groups, such as -OH groups, for acryloyl group synthesis for further polymerization.

In this study, three PCL-based acrylates were synthesized. The first prepolymer is a linear molecule with two acrylates at each end, named polycaprolactone-530 diacrylate (PCL530DA). In addition, two branched molecules, glycerol-3 caprolactone triacrylate (Glycerol-3CL-TA) and glycerol-6 caprolactone triacrylate (Glycerol-6CL-TA), were also prepared through ring­opening polymerization of *ε*-caprolactone in the presence of glycerol, followed by acrylation reactions. The material properties of these prepolymers and their mixtures before and after photopolymerization were further evaluated. The photoinitiator, tertiary amine, and light source used for this study are camphorquinone (CQ), dimethylaminoethyl methacrylate (DMAEMA), and 470 nm blue LED light, respectively.

## 2. Material and Methods

### 2.1. Synthesis of PCL530-Diol, Glycerol-3 Caprolactone Triol, and Glycerol-6 Caprolactone Triol

For the synthesis of PCL530 diol, diethylene glycol was used as the starting material. Diethylene glycol was dried at 130°C for 1 hour; then, an appropriate amount of *ε*-caprolactone at a 1 : 4 molar ratio was polymerized in bulk in the presence of diethylene glycol under a nitrogen atmosphere at room temperature for 24 hours by using 1% stannous octoate as the catalyst. For synthesis of glycerol-3 caprolactone triol and glycerol-6 caprolactone triol, glycerol was dried and thoroughly mixed with *ε*-caprolactone at a 1 : 3.3 and 1 : 6.6 molar ratios, respectively. The mixture was then heated to 130°C and interfacial polymerization occurred for 24 hours.

### 2.2. Synthesis of PCL530DA, Glycerol-3CL-TA, and Glycerol-6-CL-TA

**Figure 1:**
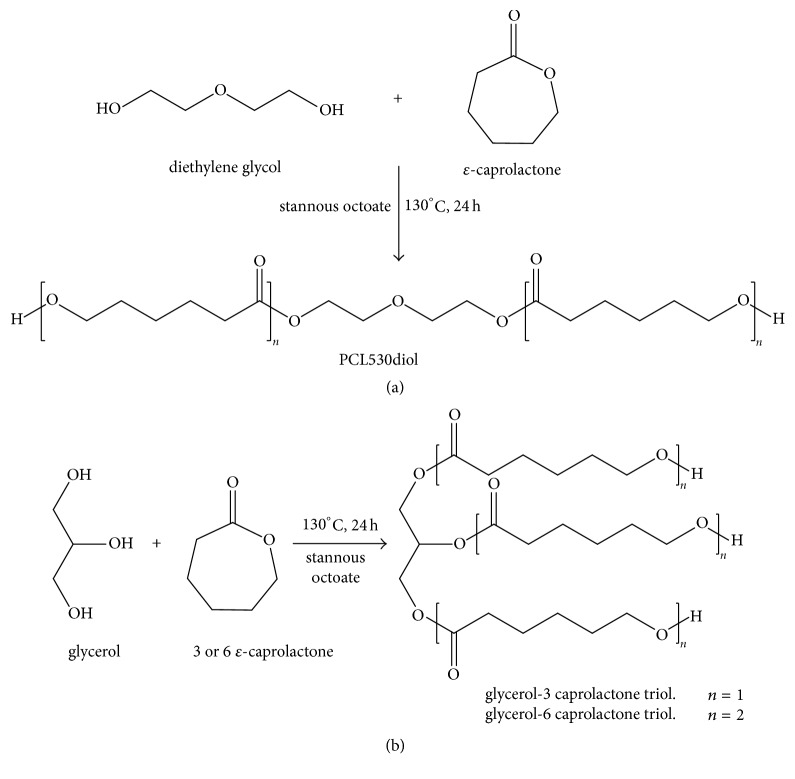
Synthesis procedure for PCL530 diol (a), glycerol-3 caprolactone triol, and glycerol-6 caprolactone triol (b).

**Figure 2:**
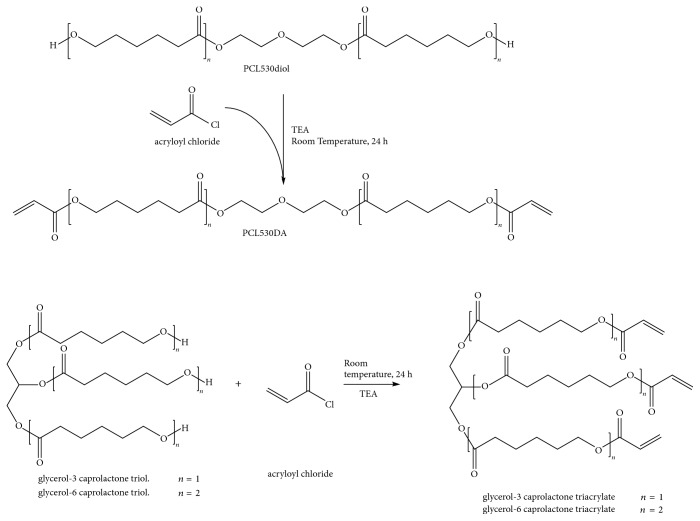
Synthesis procedure for PCL530DA, Glycerol-3-CL-TA, and Glycerol-6-CL-TA.

PCL530DA, Glycerol-3CL-TA, and Glycerol-6CL-TA were prepared by the reaction of acryloyl chloride with PCL530-diol, Glycerol-3 caprolactone triol, and Glycerol-6 caprolactone triol, respectively (Figures 1 and 2). PCL530 diol, glycerol-3 caprolactone triol, and glycerol-6 caprolactone triol were first dried at 120°C under a nitrogen atmosphere for 1 hour and then cooled down to less than 50°C. They were then dissolved in tetrahydrofuran, and trimethylamine (Sigma-Aldrich, USA) was added to the solution. Acryloyl chloride (Merck KGaA, Darmstadt, Germany) at a 1 : 2.2 (for PCL530DA) or 1 : 3.3 (Glycerol-3CL-TA or Glycerol-6CL-TA) molar ratio was slowly added to the solution. The reaction mixture was stirred continuously for 24 hours in the dark at room temperature under a nitrogen atmosphere. The solvent was removed overnight using a rotary evaporator. The reaction product was stored in the dark at room temperature.

### 2.3. Fourier Transform Infrared Absorption Spectrum of the PCL-Based Acrylates

The infrared (IR) spectra were used to determine whether the materials were well synthesized. The chemical bonds of the specimens tested could be found and are shown as peaks on the diagram. All specimens were evaluated using a Nicolet™ iS5TM Fourier transform infrared spectrometer (ThermoFisher, MA, USA) equipped with an attenuated total reflectance (ATR) device comprising a horizontal ZnSe crystal. The samples prepared were large enough to cover the whole surface of the ATR crystal and were scanned at least 16 times. Collected spectra were acquired in the range of 800–4000 cm^−1^, with a resolution of 4 cm^−1^.

### 2.4. ^1^H Nuclear Magnetic Resonance Spectroscopy

Chemical bonds could be investigated through the IR spectra, but the peaks may be overlaid on the diagram to affect the interpretation of the results. The quantity of each element in a specimen could be determined through ^1^H-nuclear magnetic resonance (NMR) spectroscopy. For the NMR measurement, 10 mg of samples was dissolved in 1 ml of deuterated chloroform with tetramethylsilane as an internal standard. ^1^H-NMR spectra were acquired at a temperature of 300 K using a Bruker Ascend 400-MHz spectrometer. Each specimen was scanned 32 times to obtain an average spectrum. Chemical shifts were referenced relatively to chloroform at 7.26 ppm in the ^1^H-NMR spectra. Peaks on the spectra were delimited and integrated on Topspin (version 3.0).

### 2.5. Viscosity of the PCL-Based Acrylates

Viscosity is a critical property of 3D printing materials. Therefore, the viscosity of PCL530DA, Glycerol-3CL-TA, and Glycerol-6CL-TA was measured with a cone-plate viscometer (Brookfield DV3TRV Rheometer, Wells-Brookfield Cone/Plate; Brookfield Engineering Laboratories Inc., Middleboro, MA, USA) at room temperature. To determine the best compromise between viscosity and mechanical properties of the materials after photopolymerization, the viscosity of the materials mixtures was further investigated. A constant volume (1 cm^3^) of each resin was dispensed between the cone and plate, and the viscosity was collected at 10 rpm with a shear rate of 38.4 s^−1^ for 2 min. The data was collected every second, and the readings between 10 and 95% torque were recorded and expressed in centipoise (cps).

### 2.6. Determination of the Degree of Conversion of PCL-Based Acrylates

To estimate of the degree of conversion (DC) of the PCL-based acrylates, five light-cured resin solutions were exposed to 470 nm LED blue lights (6 mW cm^−2^) for 10, 20, 30, and 60 s. The IR spectra of the light-cured resins were obtained using the same Fourier transform IR (FTIR) spectrometer. Before calculating of the DC of the samples, all sample spectra were normalized to the C=O peak at 1720 cm^−1^ because the number of the carbonyl bonds did not change after the light induced polymerization. The DC for each sample was then determined by comparing the intensity of the aliphatic C=C stretching vibration at 1640 cm^−1^ of the polymerized resin and the negative control, the unpolymerized resin. DC (%) was calculated by subtracting the percentage of remaining aliphatic C=C from 100%.

### 2.7. Compression Test of the PCL-Based Acrylates

All three PCL-based acrylates containing 1.2% CQ and 2.4% DMAEMA were prepared and light-cured under 6 mW cm^−2^ for 1 min. A universal testing machine (Shimadzu AGS-500G) equipped with a 100 kg or 500 kg load cell in compression mode was used to measure the compressive strength and modulus of the samples at room temperature. Light-cured samples with a diameter of 14 mm and a thickness of 8 mm were used. All tests were conducted until the samples were crushed. The crosshead speed was 2 mm min^−1^. To obtain statistically reliable results, all measurements were repeated at least three times.

### 2.8. Statistical Analysis

Experimental data was processed using on GraphPad Prism (version 6). Quantitative data were analyzed using one-way analysis of variance. The data are presented as mean ± standard deviation. A *p* of <0.05 was considered statistically significant.

## 3. Results

### 3.1. FTIR and NMR Spectrum

**Figure 3:**
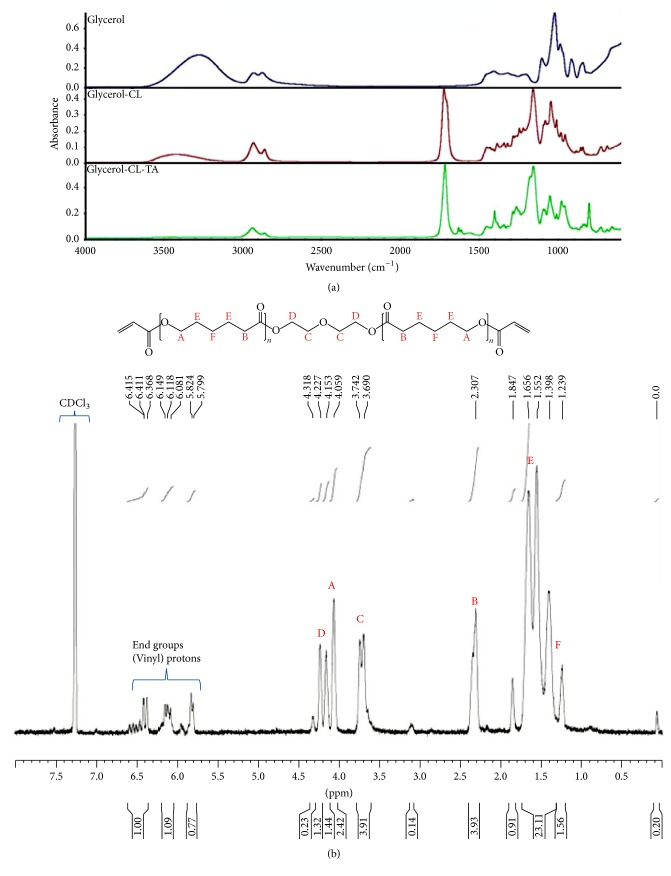
(a) FTIR spectra of Glycerol-3CL-TA at different steps of the synthesis. Top: glycerol; middle: Glycerol-3CL triol; bottom: Glycerol-3CL-TA. (b) NMR spectrum of Glycerol-3CL-TA. Three groups of peaks within 5.8–6.5 ppm correspond to vinyl protons in the acrylate groups.

**Figure 4:**
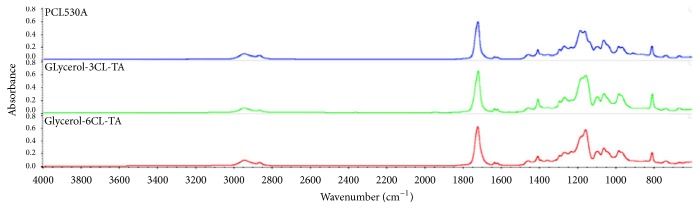
FTIR spectrum of the three PCL acrylates, including PCL530DA (top), Glycerol-3CL-TA (middle), and Glycerol-6CL-TA (bottom).

Figure 3(a) shows the FTIR spectra of the starting materials glycerol (top), the intermediate Glycerol-3CL triol (middle), and the final product Glycerol-3CL-TA (bottom) during the synthesis. Strong C=O bonds at 1730 cm^−1^ can be seen in the spectra for Glycerol-3CL triol and Glycerol-3CL-TA, suggesting the presence of -C=O from *ε*-caprolactone. Small vinyl C=C peaks can be found at 1640 cm^−1^ in Glycerol-3CL-TA, suggesting the presence of the acrylates at both ends. The -OH peaks in 3300 cm^−1^ drop during the synthesis, as shown in Figure 3. The decrease of -OH groups was in agreement with the synthesis steps shown in Figure 2. This suggests that the -OH groups were adapted by the acrylate functional groups.  Figure 3(b) shows the NMR spectrum of the Glycerol-3CL-TA. Three groups of peaks within 5.8–6.5 ppm correspond to vinyl protons in acrylate, suggesting the presence of acrylate groups.  Figure 4 shows the FTIR peaks of the PCL530DA (top), Glycerol-3CL-TA (middle), and Glycerol-6CL-TA (bottom). Small vinyl C=C peaks can be found at 1640 cm^−1^ in all PCL acrylates.

### 3.2. Viscosity

**Figure 5:**
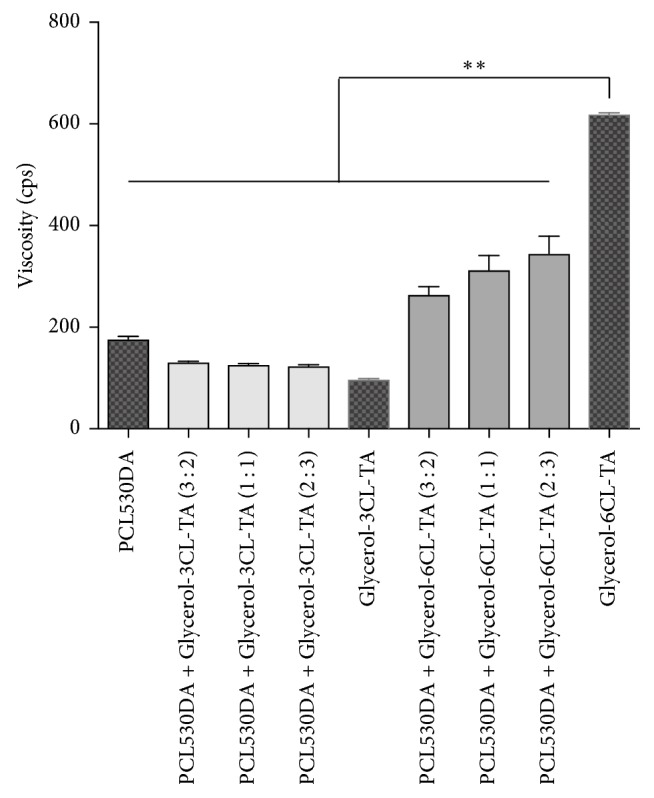
Viscosity of PCL530DA, Glycerol-3CL-TA, Glycerol-6CL-TA, and their mixtures at 3 : 2, 1 : 1, and 2 : 3 ratios. ^*∗∗*^*p* < 0.01.

At room temperature, PCL530DA is a transparent liquid, with a viscosity of 176.8 cps (Figure 5). Both Glycerol-3CL-TA and Glycerol-6CL-TA are also liquids at room temperature, with viscosities of 97.7 and 619.3 cps, respectively (Figure 5). When PCL530DA is mixed with Glycerol-3CL-TA or Glycerol-6CL-TA, the viscosity of the resulting mixtures falls within the viscosities of the constituent prepolymers.

**Figure 6:**
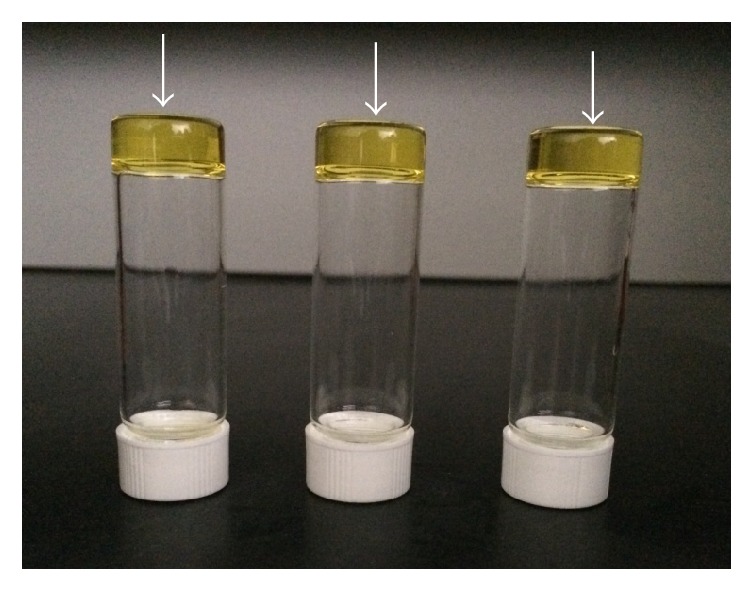
The arrows point to the yellowish materials identified as photopolymerized Glycerol-6CL-TA, Glycerol-3CL-TA, and PCL530DA after curing (left to right).

**Figure 7:**
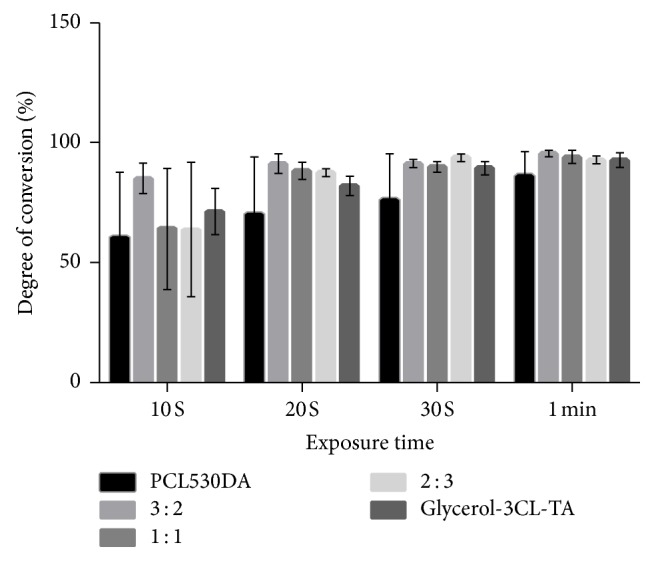
DC of PCL530DA, Glycerol-3CL-TA, and their mixtures at 3 : 2, 1 : 1, and 2 : 3 ratios under blue light exposure.

**Figure 8:**
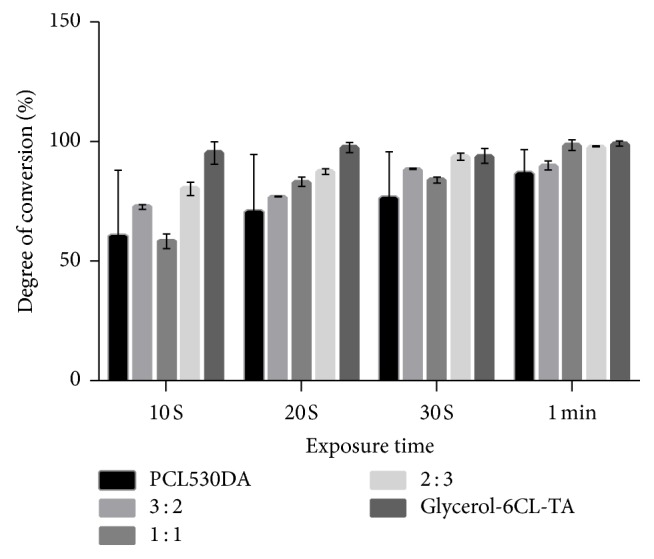
DC of PCL530DA, Glycerol-6CL-TA, and their mixtures at 3 : 2, 1 : 1, and 2 : 3 ratios under blue light exposure.

After light exposure, all PCL acrylates became transparent solids (Figure 6). The DC of the PCL acrylates and their mixtures was evaluated by comparing the height of the C=C peak of the light-cured resin with that of the uncured resins (Figures 7 and 8). After 10 s of exposure to blue light, all resins showed a DC > 50%. When the curing time increased from 10 to 60 s, all samples reached 80% DC. Notably, the DC of Glycerol-6CL-TA was constant between 10 and 60 s curing time.

### 3.3. Compressive Test

**Figure 9:**
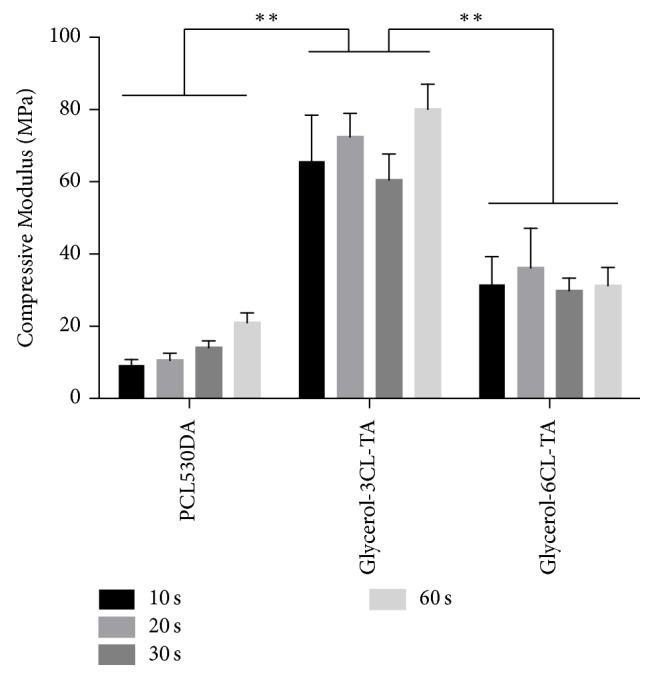
Compressive modulus of PCL530DA, Glycerol-3CL-TA, and Glycerol-6CL-TA for different curing times. ^*∗∗*^*p* < 0.01. The compressive modulus of Glycerol-3CL-TA is significantly higher than for PCL5300DA and Glycerol-6CL-TA at all curing times tested.

**Figure 10:**
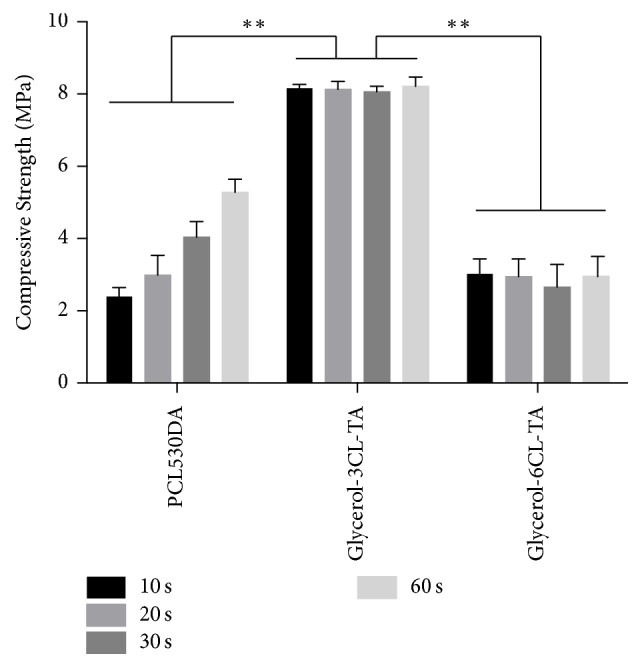
Compressive stress of PCL530DA, Glycerol-3CL-TA, and Glycerol-6CL-TA for different curing times. ^*∗∗*^*p* < 0.01. The compressive strength of Glycerol-3CL-TA is significantly higher than for PCL5300DA and Glycerol-6CL-TA at all curing times tested.

After curing under blue light, the compression tests showed that PCL530DA and Glycerol-6CL-TA had a much lower compressive modulus and compressive strength than Glycerol-3CL-TA for all curing times tested (Figures 9 and 10). In addition, the compressive modulus of both PCL530DA and Glycerol-6CL-TA seemed to increase proportionally to the compressive strength as the curing time increased.

## 4. Discussion

In this study, three PCL prepolymers, namely, PCL530DA, Glycerol-3CL-TA, and Glycerol-6CL-TA, were prepared. All these prepolymers have a common characteristic: their viscosity is smaller than 800 cps—important for 3D printing materials. A smaller viscosity enables the molecules to flow and separate efficiently in the resin tank, which makes the prepolymers suitable for most commercial DLP and SLA 3D printers. This low viscosity was similar to that of commercially available 3D printing resins from Formlabs, the 3D printing company with the largest market share.

PCL530DA has two acrylates in its structure, whereas Glycerol-3CL-TA and Glycerol-6CL-TA have three. During photopolymerization, prepolymers that have at least two acrylates can form a cross-linked network. In other words, all three prepolymers studied can form a cross-linked network after exposure to light. Nevertheless, the DC of 100% PCL530DA is lower at all curing times compared with (1) Glycerol-3CL-TA, (2) Glycerol-6CL-TA, and (3) the mixture of PCL530DA with Glycerol-3CL-TA or Glycerol-6CL-TA. This can be caused by the lower density of acrylate in the linear PCL530DA. The DC of Glycerol-3CL-TA increased rapidly during 10–20 s light exposure. Then, it increased in a flat slope until 60 s curing time. By contrast, Glycerol-6CL-TA retained a high DC from 10 to 60 s. The rate of the DC is determined by the frequency of an acrylate to meet another acrylate [27, 28]. When a molecule is longer, the acrylate at the end of the molecule has a higher chance to react with the acrylate from another molecule. If the DC of a resin prepolymer is too low after light exposure, the unreacted elements within the resin molecule can diffuse out of the resin into the surrounding environment, resulting in cell death during cell culture tests. Therefore, DC is an important indicator for a light-cured resin.

DC also plays an important role for the mechanical properties of materials [29–32]. To understand the compressive properties of these materials in their fully cured state, the PCL prepolymers polymerized after 1 minute of exposure to light were tested once they had reached their highest DC. The compressive modulus and compressive strength of photopolymerized Glycerol-3CL-TA are significantly higher than those of Glycerol-6CL-TA, although Glycerol-6CL-TA has a longer polymer chain and a higher viscosity before photopolymerization. This is because at the same weight, Glycerol-3CL-TA contains a higher acrylate density than does Glycerol-6CL-TA. Therefore, a higher cross-linking density can be achieved after exposure to light. This explains the reason that Glycerol-3CL-TA has better compressive properties. The compressive modulus (80.9 ± 6.1 MPa) and compressive strength (8.3 ± 0.18 MPa) of Glycerol-3CL-TA are too low for bone tissue engineering applications. However, it can still be used for soft tissue engineering or bone regeneration where mechanical loading is not a concern (e.g., extraction sockets).

## 5. Conclusions

In this study, three PCL-based photopolymerizable materials were prepared. Because these materials can be light-cured to form a solid and have low viscosity, they have great potential for soft tissue engineering and medical 3D printing.
